# Fast Online Coordinate Correction of a Multi-Sensor for Object Identification in Autonomous Vehicles

**DOI:** 10.3390/s19092006

**Published:** 2019-04-29

**Authors:** Wooyoung Lee, Minchul Lee, Myoungho Sunwoo, Kichun Jo

**Affiliations:** 1 Autonomous Driving Platform Team, Hyundai Motor Company, Seoul 06797, Korea; ericlee0829@hyundai.com; 2 Department of Automotive Engineering, Hanyang University, Seoul 04763, Korea; minchul.baker.lee@gmail.com (M.L.); msunwoo@hanyang.ac.kr (M.S.); 3 Department of Smart Vehicle Engineering, Konkuk University, Seoul 05029, Korea

**Keywords:** mutli-sensor cooirdinate matching, data association, object identification, online parameter estimation, multi-sensor object convergence, autonomous vehicle

## Abstract

Multi-sensor perception systems may have mismatched coordinates between each sensor even if the sensor coordinates are converted to a common coordinate. This discrepancy can be due to the sensor noise, deformation of the sensor mount, and other factors. These mismatched coordinates can seriously affect the estimation of a distant object’s position and this error can result in problems with object identification. To overcome these problems, numerous coordinate correction methods have been studied to minimize coordinate mismatching, such as off-line sensor error modeling and real-time error estimation methods. The first approach, off-line sensor error modeling, cannot cope with the occurrence of a mismatched coordinate in real-time. The second approach, using real-time error estimation methods, has high computational complexity due to the singular value decomposition. Therefore, we present a fast online coordinate correction method based on a reduced sensor position error model with dominant parameters and estimate the parameters by using rapid math operations. By applying the fast coordinate correction method, we can reduce the computational effort within the necessary tolerance of the estimation error. By experiments, the computational effort was improved by up to 99.7% compared to the previous study, and regarding the object’s radar the identification problems were improved by 94.8%. We conclude that the proposed method provides sufficient correcting performance for autonomous driving applications when the multi-sensor coordinates are mismatched.

## 1. Introduction

In autonomous driving systems, one of the most important factors to plan and control a vehicle’s motion is the understanding of the autonomous vehicle’s environment, especially when moving objects approach it. To obtain this information, various perception sensors such as the camera [1,2,3], lidar [4,5,6], and radar [7,8] have been developed. However, single sensor-based perception systems have performance constraints for recognizing surrounding moving objects because of sensor limitations. For example, the camera can make a better estimation of the lateral position and classification of the moving object than radar and lidar can. On the contrary, the radar and lidar can make a better estimation of the longitudinal distance of a moving object whilst have a longer detection range than the camera. To combine the advantages of each sensor, multiple sensor-based perception systems are widely used in autonomous driving systems [9,10,11,12,13,14].

**Figure 1:**
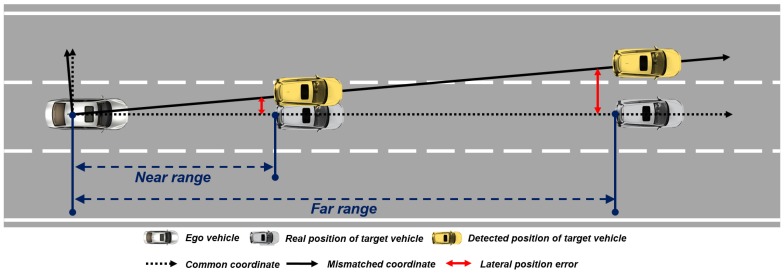
The serious effect of the mismatched coordinate to the distant object.

To obtain the moving object’s information from the multi-sensor, converting the coordinates of each sensor into a common coordinate must be preceded, as each sensor has a different installation position, type of coordinate, and measuring units. In this paper, we define the coordinate conversion process as rotating and translating the sensor coordinate for matching into a common coordinate based on origin, orientation difference between sensors and a common coordinate. Even if the coordinate conversion process is completed, the sensor coordinate may slightly mismatch, due to the sensor’s noise effect, the sensor’s mount deformation due to aging, and the dynamic movements of the vehicle. The slightly mismatched coordinate causes the position error of moving objects from each sensor. Moreover, as a target object moves further, the position error becomes larger. For example, if the azimuth orientation does not match 1-degree, a 0.17 m lateral position error can occur for an object 10 m away, however, a 1.7 m lateral position error occurs for an object 100 m away, as shown in Figure 1. In other words, even if multi-sensor detects the same distant object, it is difficult to determine as the same object due to lateral position error due to the coordinate mismatch. We name this an object identification problem.

**Figure 2:**
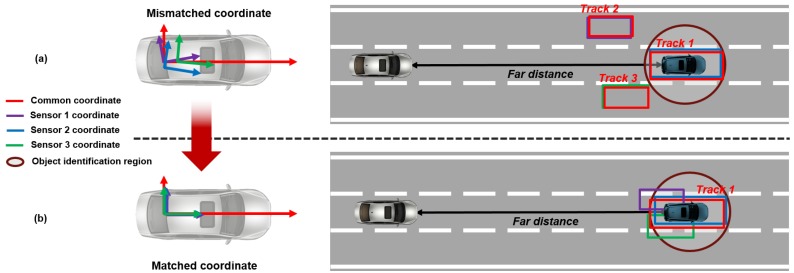
The concept of coordinate correction to solve the object identification problem. (**a**) The object identification problem caused by coordinate mismatching. (**b**) The object identified by coordinate correction.

The object identification problem can cause malfunction of the autonomous driving system, such as collision avoidance and lane change applications, because the system can possibly determine that many objects exist in the frontal area and there is no place to avoid or move, as shown in Figure 2a. Thus, obtaining reliable moving object information by correcting the mismatched coordinate is very important to the autonomous driving system, as shown in Figure 2b. In addition, since the sensor is installed in the sensor mount, coordinate misalignment can occur in real-time by deformation on the sensor mount with the time-lapse and light collision. Furthermore, variation in the pitch and the roll of the vehicle arising from the dynamic driving environment can occur in the slight coordinate mismatching between sensors in real-time. Therefore, mismatched coordinate between multi-sensor must be corrected in real-time to obtain the reliable moving object information from multiple sensors.

There are many approaches concerning coordinate misalignment. One of the approaches uses off-line sensor error modeling based on RTK-GPS [15,16]. The off-line sensor error modeling attempts to solve the misaligned coordinate by modeling the position error of sensor measurements and RTK-GPS information. This method has the advantage to obtain accurate reference positions from RTK-GPS. Another advantage is that the corrected object information could be obtained without an additional computation effort to estimate the model parameters since the model parameters are already estimated off-line. However, this method requires an expensive equipment, the RTK-GPS, to estimate the object information error and could not cope with the coordinate mismatch occurring in real-time. Another approach is minimizing the position error between sensor measurements based on the error estimation methods, such as iterative closest point (ICP), least square method (LSM), amongst other methods [17,18,19,20,21]. One of the previous studies in this approach uses lidar, which has a position accuracy of less than 0.1m, as the reference sensor and correcting the other sensors with ICP algorithm. This method has an ability and advantage to handle the online coordinate mismatching problem. Although this method is effective, this method requires a high computational effort to calculate the singular value decomposition (SVD), which, is used in ICP. Moreover, additional methods are needed to reduce the sensor noise effect.

Therefore, we propose a fast online coordinate correction method with the reduced computational effort for matching the mismatched sensor coordinates. The proposed method simplifies a sensor position error model and model parameter estimation based on using rapid mathematical operations. In this paper, the rapid mathematical operations represent the arithmetic operations and inverse trigonometric functions which take a small computational burden. The sensor position error model is designed only with the key factors of position error to reduce the model’s complexity within the necessary tolerance of model accuracy. The model parameter estimation method is used as a projection approach with recursive least square to reduce the computation complexity and noise effect within necessary tolerance of correction error.

The fast online coordinate correction method is explained in the following four sections. Section 2 describes the design for sensor position error model. This section contains the dominant parameters selection method based on performance impact analysis of coordinate mismatching factors. In Section 3, the projection approach with recursive least squares is proposed for online model parameter estimation and this section explains the synergy effects of using projection approach and recursive least squares. The proposed method is verified through vehicle experiments in Section 4. Finally, the advantages of the fast online coordinate correction method are summarized in Section 5.

## 2. Sensor Position Error Model

The coordinate conversion process is translating and rotating the sensor coordinates to match the common coordinate based on the errors of six degrees-of-freedom (DOF) parameters. Since sensors are installed in the mount, the six DOF parameters can be defined as the coordinate origin position(*x_o_*, *y_o_*, *z_o_*) of the sensor mount and the orientation(*θ_pitch_*, *θ_roll_*, *θ_yaw_*) of coordinate of the sensor mount. This setup means that the sensor position error model should be designed with errors of six DOF parameters. However, the sensor position error model does not need to consider all six DOF parameters because the object identification problem is closely related to the longitudinal, and lateral positional errors between measurements. In other words, the parameters, which do not affect the longitudinal and lateral position of a moving object, are not necessary to consider in the model’s design. Thus, the proposed sensor position error model was designed with only the dominant parameters which influence the longitudinal and lateral position changes of moving objects among the six DOF parameters. Moreover, simplifying the sensor position error model has the ability to reduce the model’s complexity.

The dominant parameters were selected through performance impact analysis, which determines the parameters that have a large influence on the longitudinal and lateral position of the moving object, by applying the offset to six DOF parameters. In the performance impact analysis, a 1 m origin offsets were given to the *x*, *y*, and *z*-axis, and 1-degree orientation offsets were given to the *θ_pitch_*, *θ_roll_*, and *θ_yaw_*, respectively. Equations for identifying the longitudinal and lateral direction position changes of a moving object by applying an offset to the six DOF parameters are as follows [22]:

(1) $$\left[\begin{array}{c} x_{m}\\ y_{m}\\ z_{m} \end{array}\right]=\left[\begin{array}{c} x_{o}\\ y_{o}\\ z_{o} \end{array}\right]+\left[\begin{array}{c} x_{offset}\\ y_{offset}\\ z_{offset} \end{array}\right]$$

(2) $$\left[\begin{array}{c} x_{m}\\ y_{m}\\ z_{m} \end{array}\right]=\left[\begin{array}{ccc} cos\theta_{pitch} & 0 & sin\theta_{pitch}\\ 0 & 1 & 0\\ -sin\theta_{pitch} & 0 & cos\theta_{pitch} \end{array}\right]\left[\begin{array}{c} x_{o}\\ y_{o}\\ z_{o} \end{array}\right]$$

(3) $$\left[\begin{array}{c} x_{m}\\ y_{m}\\ z_{m} \end{array}\right]=\left[\begin{array}{ccc} 1 & 0 & 0\\ 0 & cos\theta_{roll} & -sin\theta_{roll}\\ 0 & sin\theta_{roll} & cos\theta_{roll} \end{array}\right]\left[\begin{array}{c} x_{o}\\ y_{o}\\ z_{o} \end{array}\right]$$

(4) $$\left[\begin{array}{c} x_{m}\\ y_{m}\\ z_{m} \end{array}\right]=\left[\begin{array}{ccc} cos\theta_{yaw} & sin\theta_{yaw} & 0\\ -sin\theta_{yaw} & cos\theta_{yaw} & 0\\ 0 & 0 & 1 \end{array}\right]\left[\begin{array}{c} x_{o}\\ y_{o}\\ z_{o} \end{array}\right]$$

In the above equation, $X_{m}\left[x_{m},y_{m},z_{m}\right]$ is the position information changed according to the offset given to the six DOF parameters. Also, the other parameters, $X_{o}\left[x_{o},y_{o},z_{o}\right]$ is the object’s position obtained from the sensor, $X_{offset}\left[x_{offset},y_{offset},z_{offset}\right]$ is the translation of the object in three perpendicular directions along the axis, and where $\theta_{offset}\left[\theta_{pitch},\theta_{roll},\theta_{yaw}\right]$ is the perpendicular rotation of the object along the axis.

**Figure 3:**
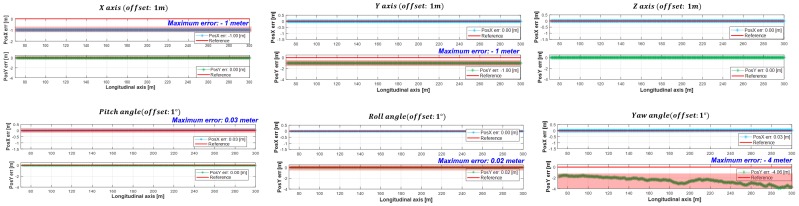
The results of the dominant factors selection by performance impact analysis.

The results of the misaligned sensor performance analysis through the above equations are shown in Figure 3. In the case of the axis offsets, the *x* and *y*-axis offsets affect to the longitudinal or lateral position. In contrast to *x*, *y*-offsets, the *z*-offset didn’t affect to the longitudinal and lateral position, as shown in the above part of Figure 3. These axis offsets could be represented by the translation matrix and (1). If the coordinate rotates 1-degree with respect to *x*-axis (the roll angle), roll angle offset affected 0.02 m to lateral position. The rotation matrix for roll angle can be represented by (3). When the coordinate rotates 1-degree based on *y*-axis (the pitch angle), it changed the 0.03 m to longitudinal position. The rotation matrix for pitch angle can be represented by (2). In the case of rotating 1-degree of *z*-axis (the yaw angle), the yaw angle was affected by up to 4 m to lateral position at 200 m away. The rotation matrix for yaw angle can be represented by (4). Excluding the z-axis offset, the offsets given to the parameters $\left(x,y,\theta_{pitch},\theta_{roll},\theta_{yaw}\right)$ affected the longitudinal and lateral position of the moving object acquired from the sensor. The effects of the offsets given to *x*, *y*, and *θ_yaw_* on the longitudinal and lateral position of the moving object were relatively larger than the effect of the offsets given to the *θ_pitch_* and *θ_roll_*. Therefore, the dominant parameters that had a large effect on the longitudinal and lateral position error of the moving object among the six degrees-of-freedom parameters were the *x* and *y*-axis origin position error and *θ_yaw_* orientation error.

The sensor position error model should be designed to reflect the dominant parameters *x* and *y*-axis origin position error and *θ_yaw_* error. The *x* and *y*- axis origin position errors could be represented by the translation of three perpendicular axes, and the *θ_yaw_* error can be represented by the rotation of the *z*-axis. Therefore, the sensor position error model could be summarized as the rotation matrix and translation matrix as shown in (5).

(5) $$\left[\begin{array}{c} x_{ref}\\ y_{ref} \end{array}\right]=\left[\begin{array}{cc} cos\theta_{err} & -sin\theta_{err}\\ sin\theta_{err} & cos\theta_{err} \end{array}\right]\left[\begin{array}{c} \left[\begin{array}{c} x_{meas}\\ y_{meas} \end{array}\right]+\left[\begin{array}{c} x_{err}\\ y_{err} \end{array}\right] \end{array}\right]$$

In the above equation, $X_{ref}\left[x_{ref},y_{ref}\right]$ is the reference position on the moving object, $X_{meas}\left[x_{meas},y_{meas}\right]$ is the object position obtained from the sensor, $X_{err}\left[x_{err},y_{err}\right]$ is the origin position error in the longitudinal and lateral, and $\theta_{err}$ indicates the $\theta_{yaw}$ orientation error.

## 3. Online Estimation of Model Parameters

As described in the introduction, parameter estimation methods such as ICP, LSM, and other methods use pseudo inverse operation to estimate the parameters of under-determined systems. The pseudo inverse matrix can be obtained by calculating the singular vector matrix with the modified singular value matrix which is inverse and transposed. Singular value decomposition(SVD) is widely used to obtain the singular value matrix and singular vector matrix from the matrix because SVD can decompose the matrix into a singular value matrix and singular vector matrix, even if the matrix is not a square matrix [23]. However, estimation methods using SVD have the property of increasing the accuracy of estimating parameters as the amount of data increases, whilst increasing the computational complexity. These methods correct the mismatched coordinates by collecting the data for a certain period of time to guarantee the reliability of estimated parameters. Thus, these methods are difficult in terms of solving coordinate mismatching problems due to the change of the position of the sensor mount, according to the vehicle dynamics occurring in real-time and require large computational resources. To overcome this problem, we proposed the projection approach with recursive least squares method, that estimates the model parameters for each time step.

### 3.1. Projection Approach with Recursive Least Squares

The projection approach with recursive least squares method rapidly calculates the orientation and origin position errors between the reference position of the moving object and the position of the sensor measurements through the projection approach. Since sensor measurements are generated under the assumption of Gaussian white noise, the states that represent the origin position and orientation error include uncertainty due to the sensor’s noise. These state uncertainty are corrected through probability-based modeling by recursive least squares.

#### 3.1.1. The Reference Object Position Selection Based on Multi-Sensor Object Convergence

Reference position selection is important when attempting to match the misaligned coordinates. In a previous research, RTK-GPS was used to obtain the reference position, however, this approach could not adjust correction parameters online because most of the objects in the real world do not equip RTK-GPS. In the other approach, lidar data were used as the reference position. However, lidar’s coordinate can also mismatch to the common coordinate. In this case, even if lidar and other sensor are matched into the same coordinate, this coordinate may still have mismatching with the common coordinate. In other words, the reference position is difficult to define with a specific sensor, such as lidar and other sensors. To overcome these limitations, we define the reference position as the position of the converged track, estimated through multi-sensor object convergence, whereby the tracks represent the result of multi-sensor object convergence. To apply the converged track as the reference position, we assumed that converged track in the near range had no difference with converged track position which the sensor coordinates are matched. This is because, in the case of the object located in close distance, misaligned coordinate affects small position error within the tolerance of sensor position accuracy. Moreover, since multi-sensor object convergence estimates the track position based on combining each sensor’s advantages, the converged track information is more reliable than track information from single sensor-based object tracking.

**Figure 4:**
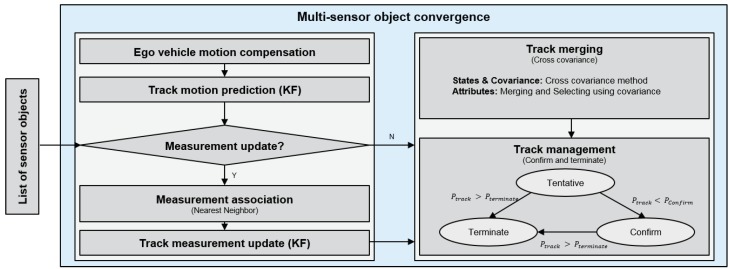
The system architecture of multi-sensor object convergence.

The details of the multi-sensor object convergence method are follows; the object data that were obtained from the multiple sensors have a position and velocity, amongst other information. Generally, the object information came from different sensors are asynchronized to a common timer and do not provide correlation information from each sensor’s internal tracking method. It is for these reasons that make it difficult to converge each sensor’s information. In order to improve the accuracy and reliability of information, the methods, nearest neighbor filter (NNF) [24] and cross covariance method (CCM) [25] based multi-sensor object convergence are applied, as shown in Figure 4. These methods are composed of track correction and track merge briefly. In the track correction step, the object data from multiple sensors are used as measurements. The NNF corrects the states and the covariance of the track by calculating the nearest measurement based on the Mahalanobis distance. In addition, the measurement must be located inside of an object identification’s region. The object identification’s region is the region for identifying the same object and generally the size of the region is obtained from inverse chi-square distribution at 0.95 or 0.99 [26]. If the measurement is not associated with the existing track, the measurement generates a new track. In the track merge step, the CCM converges tracks. Which tracks are identified as the same object, is based on the existing tracks’ status and covariance. The track management manages the status of the track and outputs the tracks which are confirmed.

#### 3.1.2. The Error Calculation Based on the Projection Approach

**Figure 5:**
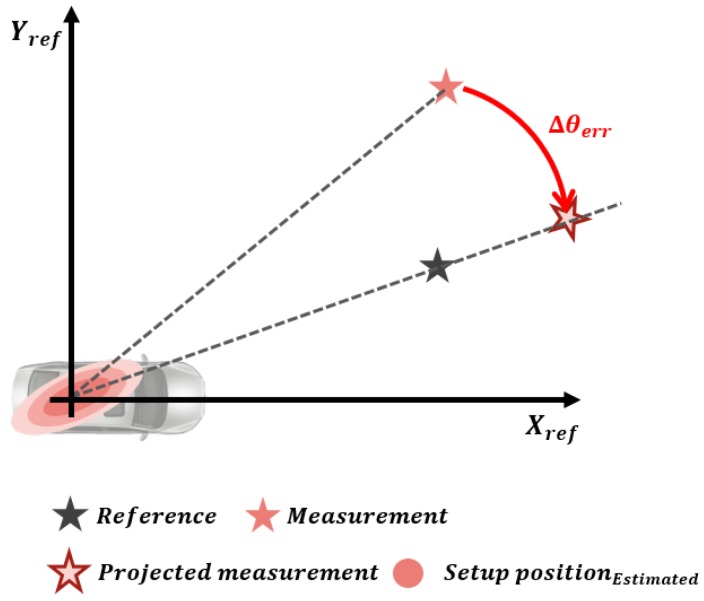
Orientation error estimated by the projection approach.

**Figure 6:**
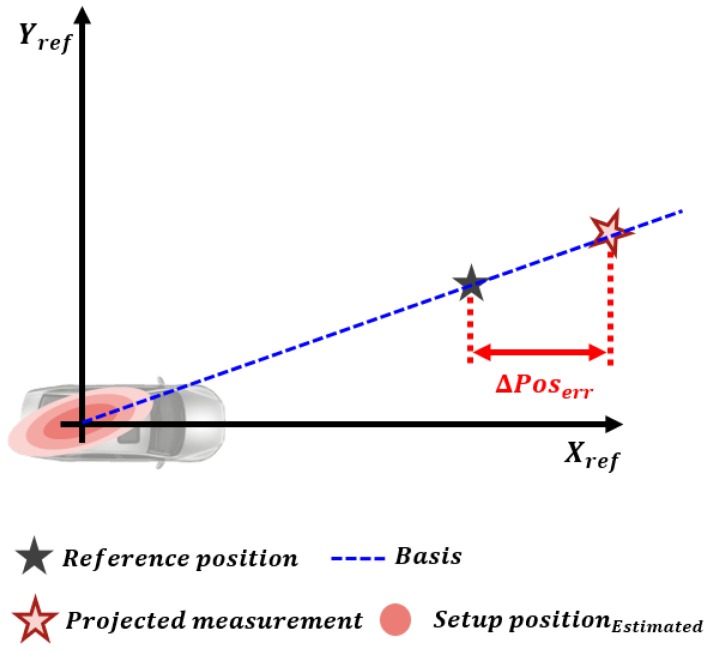
Origin position error estimated by the projection approach.

The orientation and origin position error are calculated by projection approach. The orientation error can be calculated by applying the difference between the reference and the measurement position to the inverse trigonometric function, as shown in (6) and Figure 5. As shown in (7) and Figure 6, the origin position error is the position difference between the measurement and the reference when the measurement position, including the orientation error, is projected on the line connecting the reference coordinate origin and the converged object.

(6) $$\Delta \theta_{k}=\tan^{-1}\left(\frac{y_{meas}-y_{k-1}}{x_{meas}-x_{k-1}}\right)-\tan^{-1}\left(\frac{y_{ref}}{x_{ref}}\right)-\theta_{k-1}$$

(7) $$\left[\begin{array}{c} \Delta x_{k}\\ \Delta y_{k} \end{array}\right]=\left[\begin{array}{c} x_{ref}\\ y_{ref} \end{array}\right]-\left[\begin{array}{cc} \cos\Delta \theta_{k} & -\sin\Delta \theta_{k}\\ \sin\Delta \theta_{k} & \cos\Delta \theta_{k} \end{array}\right]\left[\begin{array}{c} x_{meas}-x_{k-1}\\ y_{meas}-y_{k-1} \end{array}\right]$$

In (6) and (7), $\Delta \theta_{k},\Delta x_{k},\Delta y_{k}$ are the orientation, longitudinal, and lateral position errors between the reference and measurement at time step *k*; $\theta_{k-1},x_{k-1},y_{k-1}$ are the orientation, longitudinal and lateral position errors that have accumulated up to time step k−1; $x_{meas},y_{meas}$ are the longitudinal and lateral position coordinates of the measurement; and $x_{ref},y_{ref}$ are the longitudinal and lateral position coordinates of the reference.

The accumulated orientation and origin position error up to time step *k* can be summarized as follows:

(8) $$\theta_{k}=\tan^{-1}\left(\frac{y_{meas}-y_{k-1}}{x_{meas}-x_{k-1}}\right)-\tan^{-1}\left(\frac{y_{ref}}{x_{ref}}\right)$$

(9) $$x_{k}=\left(x_{ref}+x_{k-1}\right)+\left(x_{meas}-x_{k-1}\right)\cos\theta_{k}-\left(y_{meas}-y_{k-1}\right)\sin\theta_{k}$$

(10) $$y_{k}=\left(y_{ref}+y_{k-1}\right)+\left(x_{meas}-x_{k-1}\right)\sin\theta_{k}+\left(y_{meas}-y_{k-1}\right)\cos\theta_{k}$$

After substituting (8) into (9) and (10), the following equations are derived by applying the trigonometric addition method.

(11) $$\frac{x_{ref}+x_{k-1}-x_{k}}{\sqrt[{}]{{\left(x_{meas}-x_{k-1}\right)}^{2}+{\left(y_{meas}-y_{k-1}\right)}^{2}}}=\cos\left(\tan^{-1}\frac{y_{ref}}{x_{ref}}\right)$$

(12) $$\frac{y_{ref}+y_{k-1}-y_{k}}{\sqrt[{}]{{\left(x_{meas}-x_{k-1}\right)}^{2}+{\left(y_{meas}-y_{k-1}\right)}^{2}}}=\sin\left(\tan^{-1}\frac{y_{ref}}{x_{ref}}\right)$$

The equation of the circle in (13) is derived by applying the trigonometric identities to (11) and (12) and taking the limit of a trigonometric function.

(13) $${\left(x_{meas}-x_{k}\right)}^{2}+{\left(y_{meas}-y_{k}\right)}^{2}=x_{ref}^{2}+y_{ref}^{2}$$

**Figure 7:**
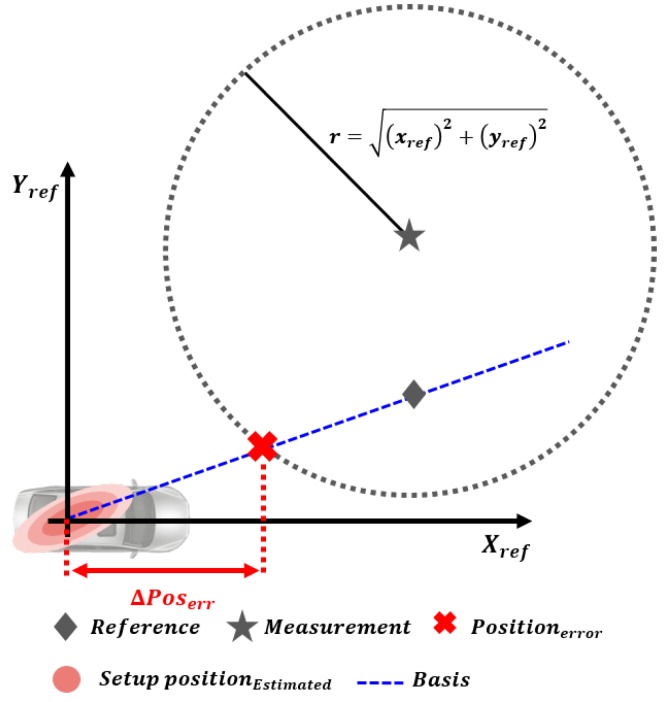
The intersection point between basis and the equation of circle.

In (13) and Figure 7, the origin position error $\left[x_{k},y_{k}\right]$ must be located at the boundary of a circle which centered at the measurement position $\left[x_{meas},y_{meas}\right]$ and with radius $\sqrt[{}]{x_{ref}^{2}+y_{ref}^{2}}$. Since the projected measurement exists on a straight line that passed the sensor coordinates origin and reference position, the intersection point of this straight line and the circle is the origin position error $\left[x_{k},y_{k}\right]$. Therefore, the straight line passing through the sensor coordinate origin and reference position is the basis on which the position error can be determined. If the basis has various positions sequentially, the states converge to the constant value.

#### 3.1.3. The Error Correction Based on Recursive Least Squares

The states are calculated by (6) and (7), which are $\Delta \theta_{k},\Delta x_{k},\Delta y_{k}$, from the measurements. However, we want to update estimated states with new measurements which are acquired sequentially and can reduce the sensor noise effect. We applied the recursive least squares method (RLS) for the state correction. If the sensor noise is considered as the constant value, RLS has no difference with the low-pass filter. However, the sensors in the autonomous vehicle, which are the camera, radar, and lidar, have performance differences depending on the detected region, such as, whether the target object is at the center of sensor’s field of view or at its boundary, it can also differ whether or not the target object is close by or far away in the distance. This performance difference can exist due to distortion and a smaller number of point clouds, and it can exist for various amount of other reasons. Therefore, we determined the RLS rather than other simple filtering methods for the noise effect reduction and optimal state estimation, by setting the large sensor noise at the boundary and far away information. The details of how we set the sensor noise differently, are based on region as described in the Appendix A. There are two advantages of using the RLS briefly. First of all, the RLS is widely used to estimate the reliable states from sequential data by stochastically modeling of the sensor noise, which can be assumed by Gaussian distribution. Secondly, RLS requires a low computational burden to estimate the states because RLS only requires the information in time step *k* − 1 and *k* [27,28]. One of the cautions to implement the RLS is outlier rejection. The outlier is the measurement that is distant to other measurements. In the case of probabilistic-based estimation, the outlier causes a decrement of estimation performance. In order to remove the outlier in the sensor measurement sets, we pre-processed the sensor measurement and applied a measurement selection. The pre-processing filters measurements based on several factors included in the acquired sensor measurement information, such as reliability value, quality level, track status, and other factors. In the measurement selection, the converged track determines the measurement based on object identification region and history of converged sensor ID. The equations for the RLS are as follows:

(14) $$K_{k}=P_{k-1}H_{K}^{T}{\left(H_{k}P_{k-1}H_{K}^{T}+R_{k}\right)}^{-1}$$

(15) $$x_{k}^{*}=x_{k-1}^{*}+K_{k}\left(y_{k}-H_{k}x_{k-1}^{*}\right)$$

(16) $$P_{k}=\left(I-K_{k}H_{k}\right)P_{k-1}$$

In (14)–(16), an estimator gain parameter represented by *K*, estimated states (estimated origin position and orientation errors) by $x^{*}={\left[\theta_{err}^{*},x_{err}^{*},y_{err}^{*}\right]}^{T}$; calculated states (calculated errors from projection approach) by $y={\left[\Delta \theta_{err},\Delta x_{err},\Delta y_{err}\right]}^{T}$; *P* is 3 by 3 matrix with uncertainty of estimated states; *I* and *H* are the 3 by 3 identity matrices; and *R* is noise covariance. The subscript *k* represents time step *k*.

The procedure of RLS is as follows: When new states from the projection approach are updated, RLS calculates the appropriate gain *K* which can be minimize the cost functions ($J=x_{ref}-x^{*}$). The gain *K* has a strong dependency on sensor noise. Refer to (14) to (16), the sensor noise affects to adjust the *K* and *P*. For instance, when the sensor noise increased, *K* become smaller and *P* become bigger than before. In other word, If the *K* is a small value, the RLS sets more weight to accumulated estimation states than the calculated states from projection approach. On the other hand, if the *K* is a big value, the RLS sets more weight to calculated errors (*x_k_*) from projection approach than accumulated estimation errors ($x_{k-1}^{*}$). Therefore, the proposed method estimates the reliable states by obtaining the new states through the projection approach and reducing the state uncertainty through RLS sequentially.

**Figure 8:**
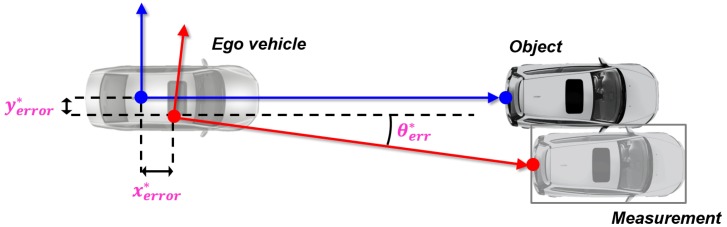
Parameters of sensor position error model.

As a results, the proposed method estimates the origin position error $\left(x_{err}^{*},y_{err}^{*}\right)$ and orientation error $\left(\theta_{err}^{*}\right)$ between the reference and sensor coordinates, as shown in Figure 8.

### 3.2. Advantages of Projection Approach with Recursive Least Squares

In this paper, we proposed the method of estimating model parameters with the reference and measurement acquired for each time step by iteratively using the projection approach and the recursive least squares. The proposed method has the advantages of reducing computational complexity, and noise effects for estimating the parameters of the under-determined system. The details are as follows. First of all, the computational complexity can be reduced by estimating the parameters of the under-determined system through the rapid mathematical operations which are arithmetic operations, and trigonometric functions. In previous studies, such as ICP, SVD is used to estimate the parameters of the under-determined system. To estimate the parameters of the under-determined system composed of *n* equations with *m* parameters through SVD, at least (*m* + 1) datasets are needed. Thus, SVD has a computational complexity of $O\left(n{\left(m+1\right)}^{2}\right)$ at a minimum. In other words, ICP increase the estimation performance and the computational complexity as the amount of datasets increases. However, the proposed method has a computational complexity of $O\left(n^{2}\right)$ because our method is performed with rapid mathematical operations. Therefore, the proposed method can estimate the parameters of the under-determined system even with the less computational resources and computation time than the previous methods. Secondly, the estimated uncertainty due to sensor noise can be reduced. Since the previous method cannot consider information uncertainty due to noise, additional filtering algorithms are necessary in order to estimate the reliable information. On the other hand, the proposed method estimates the reliable parameters by stochastically modeling the noise that has Gaussian distribution assumption through the recursive least squares method. Therefore, the influence of uncertainty, such as noise, is less than that of the previous methods.

## 4. Experimental Results

### 4.1. Experimental Environment

**Figure 9:**
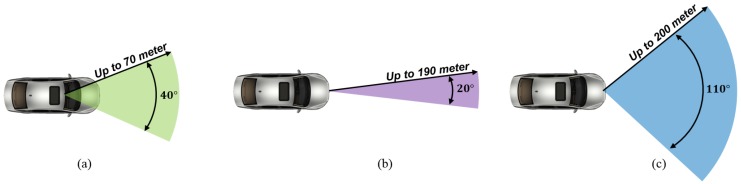
Coverage of sensors on the test vehicle: (**a**) Camera. (**b**) Radar. (**c**) Lidar.

The sensors, as shown in Figure 9, are mounted in front of the test vehicle used to verify the proposed online multi-sensor coordinate correction method. The lidar, radar, and camera were mounted on the frontal area of the test vehicle. All the sensors were conducted the coordinate conversion with respect to the vehicle coordinate which origin at the rear axle and follows the ISO coordinate.

### 4.2. Comparison between Proposed Method and ICP

To evaluate the performance of the proposed method, the ICP and the proposed method among the estimation algorithms used in a previous study [19] was compared. ICP has the following characteristics: first, the ICP is widely used for three-dimensional motion alignment. Second, the ICP can be applied to estimate the parameters of the proposed sensor position error model because ICP method estimates the rotation and translation parameters that minimizes the position error between the reference and measurement, as shown in (14).

(17) $$e=\sum_{i}{\left(\left(Rp_{i}+T-q_{i}\right)\times n_{i}\right)}^{2}$$

In (14), *R* is the rotation matrix; *T* is the translation matrix; *n_i_* is the normal vector; *p_i_* is the measurements; and *q_i_* is the reference.

**Figure 10:**
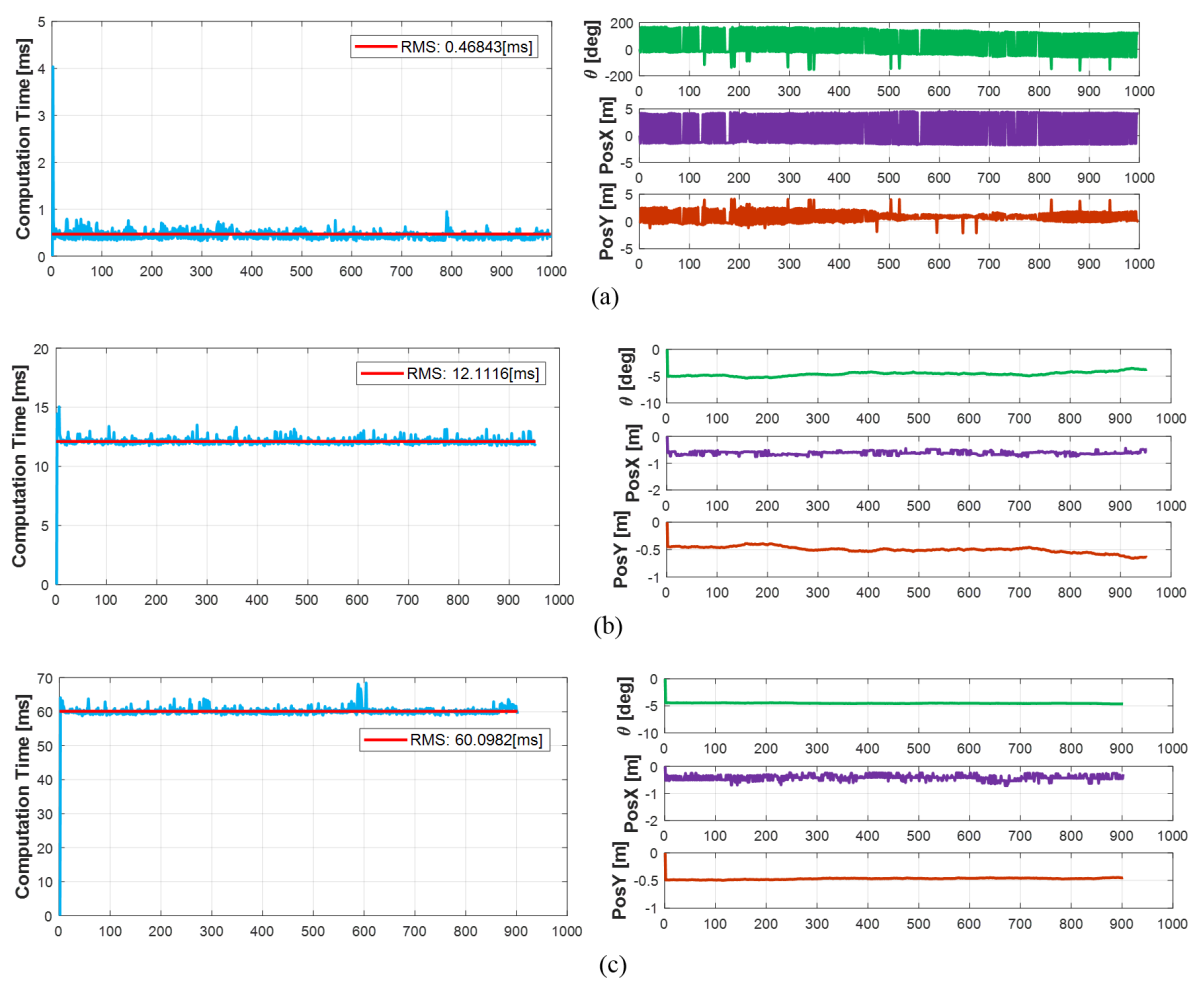
Characteristics of ICP with various datasets: (**a**) 4 datasets. (**b**) 50 datasets. (**c**) 100 data sets.

The estimation accuracy and computation time of the ICP algorithm depend on the number of datasets. In order to analyze the trade-off relationship according to estimation accuracy, the computation time and the number of datasets, we performed on the ICP with 4 datasets, 50 datasets, and 100 datasets as shown in Figure 10. The estimation accuracy of the ICP was compared with ground truth. Since the mismatch of the sensors mounted on the test vehicle could not be verified, the error between the converged track and the RTK-GPS was used as the ground truth. If the ICP estimates the parameters with 4 datasets, estimated parameters were seriously affected by sensor’s noise. From this, we can conclude that 4 datasets are not sufficient enough to use for model parameters estimation, even if, these 4 datasets have a has fast computation time of 0.47 ms. In case of 50 datasets, the ICP took 12.11 ms to estimate the parameters, the parameters’ RMS error was 0.25 m in the longitudinal direction, 0.1 m in the lateral direction, and 0.19-degrees in orientation. When performed with 100 datasets, the computation time was 60.1 ms, and the parameters’ RMS error was 0.3 m in longitudinal direction, 0.08 m in lateral direction, and 0.05-degrees in orientation. Therefore, the greater number of datasets, the slower the computation time and the better the estimation accuracy. Conversely, the smaller number of datasets, the faster the computation time and the lower the accuracy. In addition, when comparing the estimation accuracy of 50 datasets and 100 datasets, the estimation accuracy does not improve in proportion to the increase of the datasets.

#### 4.2.1. Computation Time Comparison

**Figure 11:**
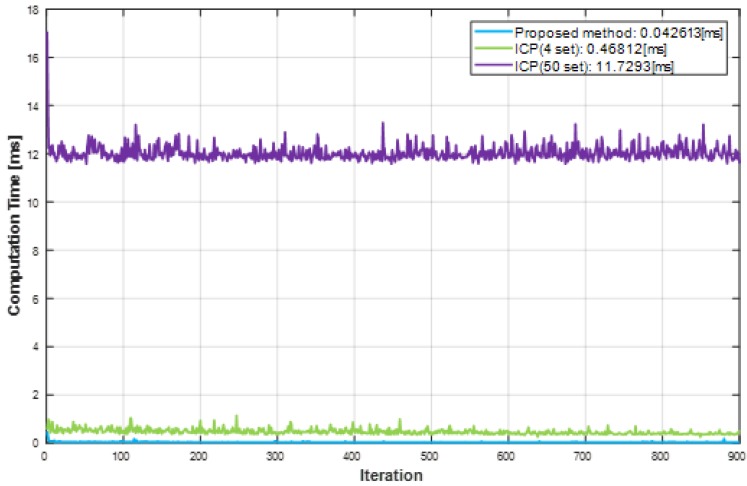
Result of computation time analysis.

To evaluate the computation time reduction using the proposed method, we compared the computation time of the proposed method and the ICP in a PC environment. We used the PC environment for comparing the computation time consisted of an Intel i5-4690 at 3.5 GHz and 8 GB memory. For the computation time comparison, the ICP was performed with 4 datasets (minimum datasets) and 50 datasets. Both methods performed a total of 900 iterations. As shown in Figure 11, the computation time of the ICP showed variation. This is because the ICP internally iterates until the error between the reference and the measurement becomes less than a certain level, thereby changing the number of iterations per dataset. As shown in Figure 11, the ICP took 0.47 ms with the 4 datasets to estimate the parameters, and 11.72 ms with the 50 datasets. On the other hand, the proposed method took 0.04 ms, therefore, the proposed method reduced the computation time at least 90.8% and up to 99.7% compared to ICP.

#### 4.2.2. Estimation Accuracy Comparison

To evaluate the estimation accuracy of the model parameters, the proposed method and the ICP were compared with ground truth. At this time, the ICP was performed with 50 datasets which have similar estimation accuracy with the proposed method. 100 datasets weren’t compared because the computation time of 100 datasets is over the sensor data update period which are among 40 ms to 50 ms as shown in Figure 10. Namely, when we applied the ICP with 100 datasets, the information delay can reduce the reliability of the object’s information, as the information is contaminated and missing. Thus, we did not compare 100 datasets performance with the proposed method. The two estimation methods performed a total of 900 iterations.

**Figure 12:**
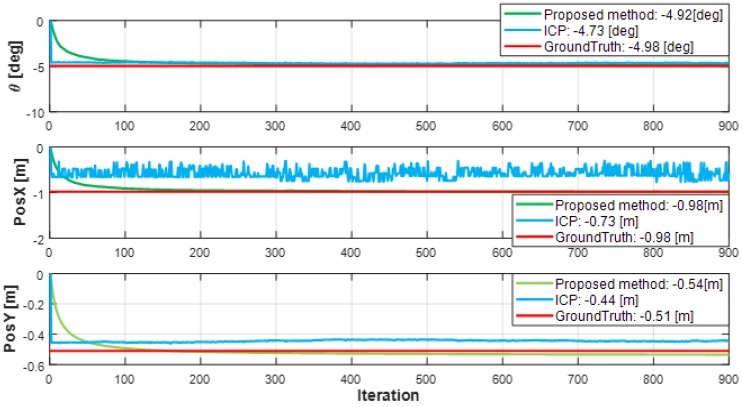
Results of model parameters estimation.

As shown in Figure 12, the proposed method estimated the state $\left[\theta_{err},x_{err},y_{err}\right]$ similarly to the ground truth. Additionally, the ICP showed variation in the estimated parameters due to sensor noise, but the proposed method was less affected by noise.

### 4.3. Effect of Online Multi-Sensor Coordinate Correction

**Figure 13:**
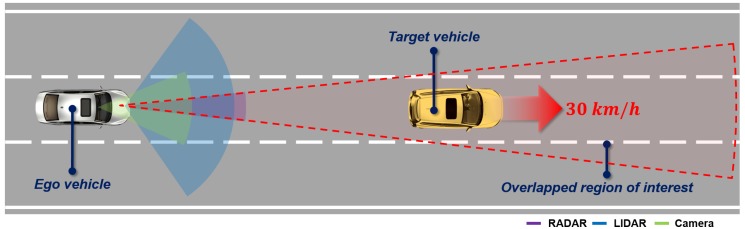
Scenario for evaluating the online multi-sensor coordinate correction.

The multi-sensor coordinate mismatch was caused by an object identification problem due to the large position error for the distant moving object. To solve this problem, the performance of the proposed multi-sensor coordinate correction method was evaluated by the position error between converged objects and sensor measurements and the status of the object identification of multi-sensor measurements. For the experiment, the scenario was composed of the target vehicle moving straight forward at 30 km/h in front of the ego vehicle to achieve information from a near to a far distance within the multi-sensors’ overlapping region of interest, as shown in Figure 13.

#### 4.3.1. Position Error between Converged Tracks and Sensor Measurements

**Figure 14:**
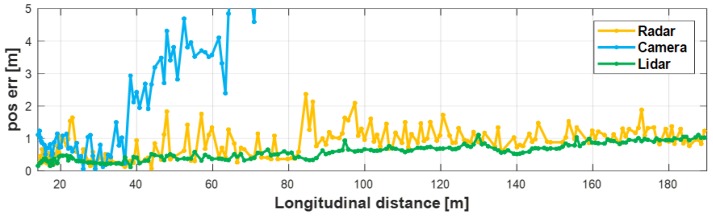
Position error when not using the proposed method.

**Figure 15:**
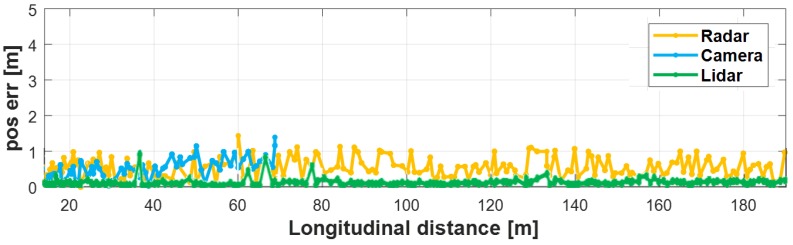
Position error when using the proposed method.

To evaluate the performance of the proposed method, we compared the position errors between the converged tracks and their measurements when the proposed method was applied and then when was not applied. As shown in Figure 14, when the proposed method was not used, all of the sensor’s object has the position error with the converged object increased with the distance from the target vehicle. If the object identification region was set to a 1 m radius, this position error could be determined as a different object from the converged tracks. However, as shown in Figure 15, the use of the proposed method significantly reduced the position error to the converged object. In the case of the radar, the distance error to the converged object was reduced within 1 m up to 190 m away; also, the camera had a distance error within 1 m from the converged object up to 70 m away; and lidar had a position error less than 0.5 m in all range. Therefore, the proposed method overcame the problem that the position error increased as the distance increased due to the slightly mismatched coordinate.

#### 4.3.2. Object Identification Status of Multi-Sensor

To evaluate the performance of the proposed method, the object identification status was compared when the proposed method was used and when the proposed method was not used. The rate of unidentified measurements was used as an evaluation index for the object identification status as shown in (18).

(18) $$\frac{\mathrm{The}\;\mathrm{number}\;\mathrm{of}\;\mathrm{unidentified}\;\mathrm{measurements}}{\mathrm{Total}\;\mathrm{number}\;\mathrm{of}\;\mathrm{detections}}\times 100\;\left(\%\right)$$

**Figure 16:**
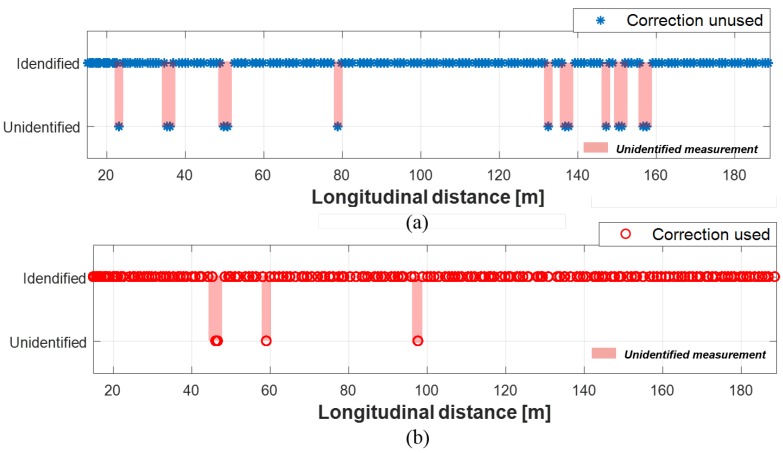
Object identification status of radar: (**a**) Without correction. (**b**) With correction.

**Figure 17:**
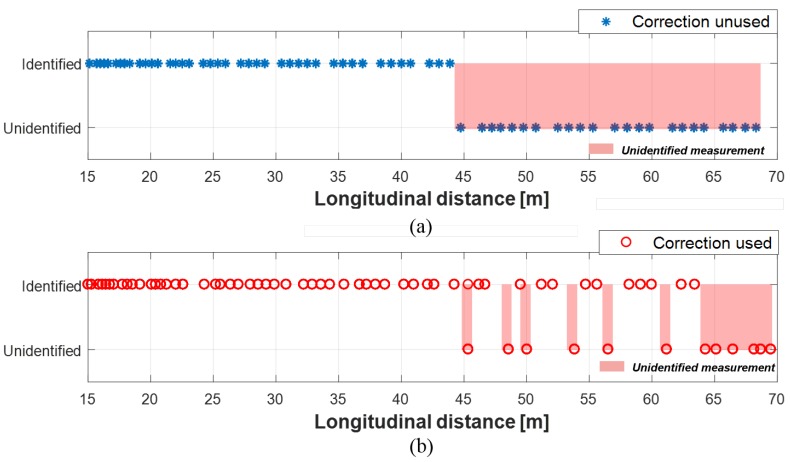
Object identification status of camera: (**a**) Without correction. (**b**) With correction.

**Figure 18:**
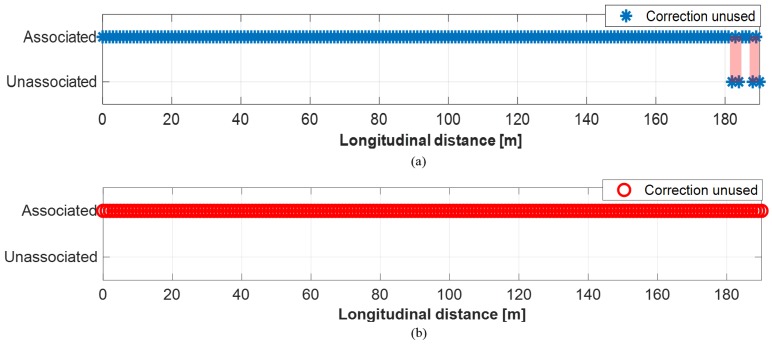
Object identification status of lidar: (**a**) Without correction. (**b**) With correction.

As can be seen in Figure 16a, 7.7% of radar measurements were misidentified when the proposed method was not applied. Likewise, as shown in Figure 17a, the measurements from camera were misidentified by 43.7% of the measurements. In the case of lidar, 0.04% of measurements were unidentified, as shown in Figure 18a. The reason for the lowest unidentification rate of lidar is that the reliability of lidar object’s position is the most reliable among the sensors, thus, the multi-sensor object convergence more depends on lidar than other sensors when the converged tracks are estimated. When the proposed method applied to these sensor measurements, the unidentification rates became significantly reduced. 0.04% of radar measurements were misidentified; 23.2% of camera measurements were misidentified; and 0% of lidar measurements are misidentified, this can be seen Figure 16b, Figure 17b, and Figure 18b. As a result, the multi-sensor coordinate correction method reduced 94.8% of misidentification in radar measurements, 46.9% of misidentification in camera measurements, and 100% of misidentification in lidar measurements.

## 5. Conclusions

This paper proposed the fast online coordinate correction method with reduced computational effort for matching the slight mismatched sensor coordinates. The advantages of this study can be summarized as follows:

The proposed method has lower computational complexity than the conventional methods, such as ICP, LSM, and other methods, because the proposed method can estimate the parameters of the under-determined system by using rapid mathematical operations, unlike previous studies that require the pseudo inverse operation or SVD. According to the experimental results, the proposed method reduced the computation time by up to 99.7% (at least 90.8%) compared with the ICP and the estimated parameters were almost the same as the ground truth. In addition, the proposed method was less affected by sensor noise than the ICP because it could probabilistically model the uncertainty of the dataset.

Since the proposed method estimates the parameters of an under-determined system using only simple mathematical operations without the pseudo inverse and SVD, the presented method can be adopted in an embedded system that does not support a floating point operation. Furthermore, a system using ICP, such as map matching, can apply the proposed method to estimate the rotation and translation parameters with faster and higher accuracy. Additionally, the proposed method can perform as an auto-calibration of heterogeneous sensors in the driver-assistance system and autonomous vehicles to maintain the reliability of estimated moving object’s information using a multi-sensor based perception system.

Although the proposed method improves the object’s identification performance of multi-sensor object convergence, it does not guarantee performance in complex environments such as crowded vehicles, which is due to the reason that the object’s identification region is set to a constant size. In order to guarantee the object’s identification performance in various environments, the authors plan to research an adaptive object identification region resizing method based on mismatched coordinate parameters. The authors also plan to integrate the proposed method into the embedded system for the feasibility test of mass production.

## Figures and Tables

**Figure A1:**
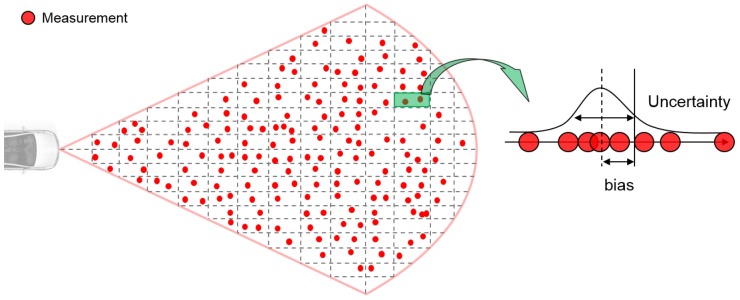
The concept of grid cells based sensor evaluation.

**Figure A2:**
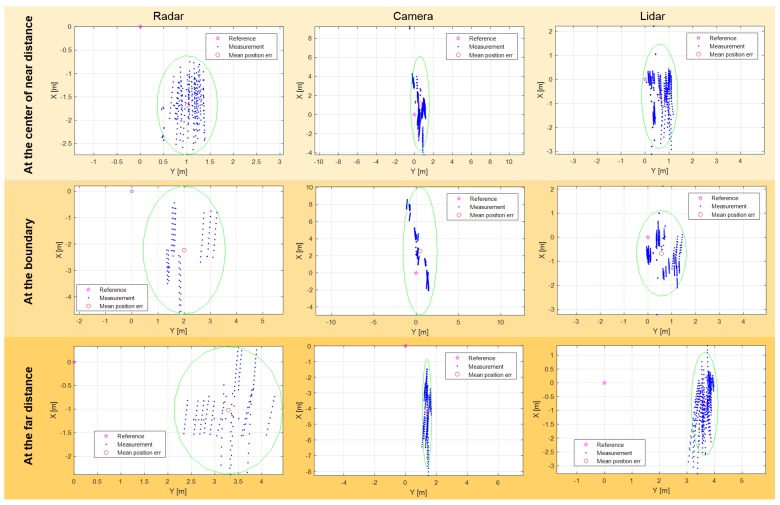
The mean position error and variance of the radar, camera, lidar at center of field of view, boundary, and far distance.

## Appendix A

Sensors have different performances depending on the detected region, therefore, we set the sensor noise differently based on detected region. To set the noise of each sensor appropriately, we performed the sensor evaluation based on grid cells. The grid cells-based sensor evaluation stacks a large amount of measurement sets to obtain reliable information; in our case, we stacked 100 measurement sets which covered the whole field of view of each sensor. After gathering the measurement sets, we divided the measurement sets into a certain size of grid cells and calculated the mean position error and variance as shown in Figure A1. The position error is the difference between reference (RTK-GPS) and sensor.

The results of grid cells based sensor evaluation, show that the sensors had a different position accuracy and variance depending on whether the target object was at the center of the field of view or at the boundary, or whether the target object was near to or far from it. In Figure A2, *X* is the longitudinal position, *Y* is lateral position, ellipsed with the green line is a variance of position error, and the red circle is mean position error. In Figure A2, all of the sensors have a small position error and variance when the target object is at the center and near in distance. If the target object went to the boundary of the field of view or far away, the mean position error and variance become larger.
